# Cochlear implantation in an animal model documents cochlear damage at the tip of the implant

**DOI:** 10.1016/j.bjorl.2020.07.017

**Published:** 2020-09-20

**Authors:** José Santos Cruz de Andrade, Peter Baumhoff, Oswaldo Laércio Mendonça Cruz, Thomas Lenarz, Andrej Kral

**Affiliations:** a Universidade Federal de São Paulo (UNIFESP), Departamento de Otorrinolaringologia e Cirurgia de Cabeça e Pescoço, São Paulo, SP, Brazil; b Coordenação de Aperfeiçoamento de Pessoal de Nível Superior (Capes), Brasília, DF, Brazil; c Institute of Audioneurotechnology (VIANNA) & Department of Experimental Otology, Department of Otolaryngology, Medical University Hannover, Hannover, Germany; d Cluster of Excellence “Hearing4all”, Hannover, Germany

**Keywords:** Cochlear implant, Hearing preservation, Guinea pigs

## Abstract

**Introduction:**

Electrocochleography has recently emerged as a diagnostic tool in cochlear implant surgery, purposing hearing preservation and optimal electrode positioning.

**Objective:**

In this experimental study, extra-cochlear potentials were obtained during cochlear implant surgery in guinea pigs. The aim was to determine electrophysiological changes indicating cochlear trauma after cochleostomy and after electrode implantation in different insertion depths.

**Methods:**

Normal-hearing guinea pigs (n = 14) were implanted uni- or bilaterally with a multichannel electrode. The extra-cochlear cochlear nerve action potentials were obtained in response to acoustic stimuli at specific frequencies before and after cochleostomy, and after introduction of the electrode bundle. After the electrophysiological experiments, the guinea pigs were euthanized and microtomography was performed, in order to determine the position of the electrode and to calculate of the depth of insertion. Based on the changes of amplitude and thresholds in relation to the stimulus frequency, the electrophysiological data and the position obtained by the microtomography reconstruction were compared.

**Results:**

Cochleostomy promoted a small electrophysiological impact, while electrode insertion caused changes in the amplitude of extra-cochlear electrophysiological potentials over a wide range of frequencies, especially in the deepest insertions. There was, however, preservation of the electrical response to low frequency stimuli in most cases, indicating a limited auditory impact in the intraoperative evaluation. The mean insertion depth of the apical electrodes was 5339.56 μm (±306.45 – 6 inserted contacts) and 4447.75 μm (±290.23 – 5 inserted contacts).

**Conclusions:**

The main electrophysiological changes observed during surgical procedures occurred during implantation of the electrode, especially the deepest insertions, whereas the cochleostomy disturbed the potentials to a lesser extent. While hearing loss was often observed apical to the cochlear implant, it was possible to preserve low frequencies after insertion.

## Introduction

The criteria for cochlear implant (CI) surgery have remarkably shifted in the last years, including patients with severe or even selective loss on mild-to-high frequencies.^1^ Improved speech perception performance scores, especially in difficult hearing conditions, in patients treated by electro-acoustic stimulation (EAS, hybrid implants) compared to patients treated by either method alone, along with the prospect of future inner ear therap, have turned the preservation of residual hearing into a critical goal in the field.^2^, ^3^ Thus minimizing trauma to the delicate intra-cochlear structures is generally an desirable objective in modern CI surgery. Recent refinements in electrode design, as well as in surgical techniques, have contributed substantially to improve postoperative hearing.^4^, ^5^ However, there is limited data and high variability between available studies on hearing preservation results. The percentage of totally or partially compromised residual hearing is still high in CI patients, despite all efforts.^6^, ^7^, ^8^

Determining the accurate electrode position as well as prompt detection of possible insertion damage is a key in current cochlear implant research. Electro-cochleography (ECochG) has recently emerged as a tool for evaluation of cochlear physiologic status and electrode insertion depth during surgery.^9^, ^10^ ECochG consists of 4 main components: compound action potentials (CAPs), a transient component at the beginning of the response commonly associated with auditory nerve activity;^11^, ^12^ auditory nerve neurophonic (ANN), which also has neural origin; cochlear microphonic potentials (CMs), an AC component throughout the stimulus response originating from the outer hair cells (OHCs);^13^ and summating potentials (SPs), a DC component in large part depending on inner hair cells (IHCs) and OHC activity.^14^, ^15^, ^16^ CAPs and CMs amplitude changes in monopolar recordings have been shown to be an early marker of contact between the electrode and cochlear structures or even minor and reversible damage, during CI insertion in gerbils.^17^, ^18^, ^19^ A correlation between intraoperative ECochG potentials and postoperative hearing preservation has been described but is still being evaluated.^20^, ^21^

The aim of the present study was to determine the site and the extent of cochleostomy and implantation trauma by documenting extra-cochlear CAP in normal-hearing guinea pigs undergoing CI surgery. The electrophysiological findings were correlated to insertion depth measured by the number of electrode channels inserted in the cochlea. We compared CAP amplitudes and thresholds in two different insertion depths in the scala tympani through cochleostomy. With this objective, we analyzed the frequencies involved in threshold shifts at different steps in the surgery and hypothesized possible causes, such as a piston effect and basilar membrane contact or damage. The intra- cochlear electrode position reconstructed from postmortem micro-computer-tomography imaging (µCT, microtomography) was checked.

## Methods

The Institutional Animal Care and Research Advisory Committee approved all experiments. The study has been conducted in accordance with the German “Law on Protecting Animals” (TierSchG) and with the European Communities Council Directive 86/609/EEC for the protection of animals used for experimental purposes.

In this study, 24 cochleae in 14 healthy and normal-hearing male Dunkin Hartley guinea pigs (Charles River Laboratories International Inc., Sulzfeld, Germany) weighing between 300 − 600 g were chosen as subjects.

## Experimental procedures

### Electrode technical details

The electrodes used in this study were custom-made research CI electrodes (Med-El Inc., Innsbruck, Austria), comprised of 6 platinum-iridium contacts spaced by 700 µm, 0.5 mm maximum diameter of the silicone carrier, tapered to 0.3 mm at the tip, and had a 4.5 mm insertable length covered by contacts (from tip to the last contact). In accordance with the standard numbering for MedEl CIs, the most apical contact will be referred as channel or contact 1 (“Ch1”), and the most basal contact as channel or contact 6 (“Ch6”), and the intermediate contacts are numbered accordingly.

### Anesthesia and monitoring

Fourteen subjects were implanted uni- or bilaterally (24 ears). All measurements and surgical procedures were performed under general anesthesia. The animals were anesthetized initially by intramuscular injection of 50 mg/kg 10% ketamine solution (CP Pharma, Burgdorf, Germany), 10 mg/kg 2% xylazine solution (WDT, Garbsen, Germany) and 0.1 mg/kg atropine sulfate (B. Braun, Melsungen, Germany). Follow-up anesthesia in 45 min’ time intervals was applied with 25%−30% of initial dose, substituting atropine sulfate by Ringer acetate solution. Anesthesia level was assessed by continuous monitoring of heart rate (using electrocardiogram) and capnometry (CO_2_%/vol.). The absence of paw-withdrawal and corneal reflexes was tested in regular intervals to verify anesthesia depth. Ample shaving of the skin behind the pinnae, around the neck, and on the vertex of the skull was performed. The animals were kept on a heating blanket (TC-1000 Temperature Controller, CWE Inc., Ardmore, USA). Core temperature was continuously monitored by a rectal probe and kept above 38 °C. A custom-made endotracheal tube was inserted through a tracheotomy and was connected to the capnometer (Normocap CO_2_ & O_2_ Monitor, Datex, Helsinki, Finland). Ventilation via endotracheal tube was performed using a rodent ventilator (Ugo Basile, Gemonio, Italy) and tidal volume was adjusted to the animal’s body weight. Respiratory rate was set between 50 and 60 per second depending on end tidal CO_2_ concentration that was kept between 3% and 4%. Animal heads were secured in a customized rodent head holder that allowed adjustment along three axes. All measures were performed in a sound-attenuated chamber.

### Auditory brainstem response (ABR) measurements

Subcutaneous Ag/AgCl electrodes were placed retroauricularly on both sides (references) of the head and at the vertex (recording electrode) for recordings. A thick Ag/AgCl electrode was subcutaneously inserted at the scruff (ground electrode). The initial hearing thresholds for both ears before surgery were determined by recording auditory brainstem responses (ABRs). A series of clicks of increasing sound levels (0 − 80 dB SPL in 5 dB steps, 50 µs click duration, 100 of each condensation and rarefaction stimulation averaged per stimulus level) were presented through a dynamic headphone speaker (DT48, Beyerdynamic, Heilbronn, Germany). The loudspeaker was positioned 1 cm away from pinna in close free-field conditions. The signals were generated, and responses acquired through the software AudiologyLab (Otoconsult, Frankfurt, Germany) via, a DA/AD converter (NI-6259, National Instruments, Austin, TX, USA). The stimuli were send through an attenuator (ATT7, Otoconsult, Frankfurt, Germany) and the recorded signals were filtered (Butterworth filter 6thorder, high-pass filter frequency 200 Hz, low-pass filter frequency 5 kHz) and amplified by 80 dB using a filter and amplifier combination (F1 device, Otoconsult Comp., Frankfurt/M., Germany). Middle ear and cochlear function were assumed normal for ABR threshold-levels of 30 dB SPL_peak equivalent_ or better. All ears included in this study satisfied this criterion.

### Surgery and cochlear implant insertion

**Figure 1:**
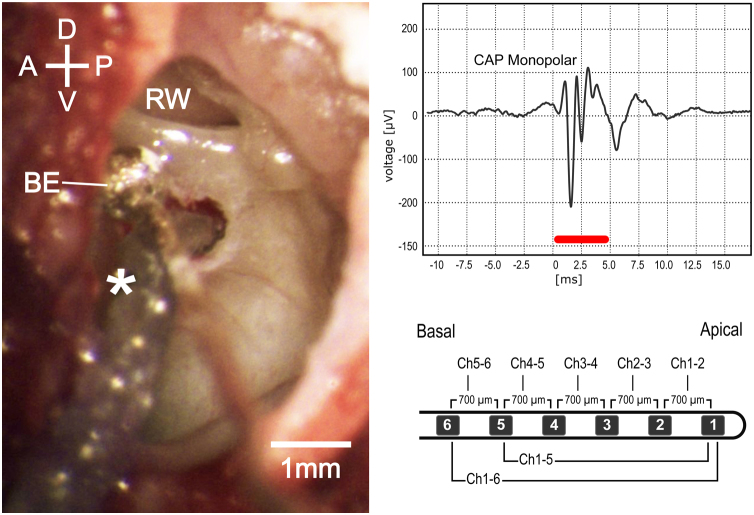
Intraoperative microscopic view (left). D, Dorsal; V, Ventral; A, Anterior; P, Posterior; be, Silver ball electrode; rw, round window. Monopolar compound action potential (upper right); red bar represents stimulus duration. Schematic image of the electrode used in experiments (lower right).

The following measurements and procedures from right or left ears were carried out sequentially (Fig. 1). Local anesthesia (2% lidocaine, 2 mL) was injected in the skin around the pinna before a complete pinna resection. Muscle and soft tissue were dissected, and the posterior-lateral part of the bulla tympani was exposed and opened with a hollow needle under stereomicroscopic visualization (Carl Zeiss OPMI® pico, Carl Zeiss, Goettingen, Germany). The lateral bulla wall was further exposed and carefully removed using a micro- rongeur to access the middle ear and the basal turn of the cochlea, including the round window (RW), without damaging the tympanic membrane. A silver ball electrode was positioned and fixed by a mechanical holder at RW inferior lip and kept in place throughout the procedure. The loudspeaker was placed near the entrance of the cartilage of the external auditory canal using a conical adapter without displacing or obstructing the ear canal. Cochleostomy was performed 0.5 mm ventral to the RW, with a 0.6 mm diamond drilling burr at 4000 rpm and care was taken to avoid bone dust or blood to enter the cochlea. The CI electrode was inserted until all 6 CI contacts were placed in the scala tympani (hereinafter referred to as “6Ch in”) or until 5 CI contacts were inserted (hereinafter referred to as “5Ch in”).

After finishing the experimental procedures, the animals were euthanatized with a transcardial infusion of 2 mL sodium pentobarbital (Release 300, WDT, Garbsen, Germany). The silastic CI carrier was then fixed in position at the rim of the tympanic bulla (Histoacryl; B. Braun Melsungen AG, Melsungen, Germany). The cadavers were decapitated, and the heads preserved in 3.5%−3.7% formaldehyde buffered solution before the following µCT imaging procedure.

### Cochlear recordings

Extra-cochlear compound action potentials (CAP) were recorded using the silver ball electrode at the RW lip. Tone bursts of 5 ms duration (2.5 ms rise/fall periods) were presented in randomized order at frequencies between 2 and 32 kHz (with logarithmic increments of 4 steps/octave), with sound pressure levels between 0 and 90 dB SPL in 10 dB steps. Stimuli were generated using the software AudiologyLab 25 (Otoconsult, Frankfurt, Germany). The sound output was calibrated using a ¼-in. microphone (type 4939, Brüel&Kjaer, Nærum, Denmark) in combination with a preamplifier (type 2670, Brüel&Kjaer, Nærum, Denmark) and a conditioning amplifier (type 2690, Nexus, Brüel&Kjaer, Denmark) connected to the National Instruments board. The recording signals were filtered between 5 Hz and 5 kHz and averaged over 20 stimulus presentations. CAP measurements were performed following the opening of the bulla, immediately after cochleostomy and after the CI insertion.

### Data analysis

Recorded data was analyzed in the range of 4 octaves (2 − 32 kHz) at 9 sound levels (0 − 80 dB_SPL_) using custom-made MATLAB routines (MathWorks, Natick, MA, USA), Prism (Graph Pad Software Inc., La Jolla, CA, USA) and Microsoft Excel (Professional Edition, 2007, Microsoft Corp, Redmond, WA). We extracted the compound action potentials (CAPs), from the electrocochleographic potential by differentially adjusted offline filters (Fig. 1). During the offline analysis of the CAPs, the recording signal was averaged over both condensation and rarefaction stimulus presentations to largely exclude CMs. The recording signal was filtered between 200 Hz and 5 kHz. CAPs were identified by the morphology of the typical N_1_ and P_1_ components at the beginning of the stimulus (analysis window between 0 and 10 ms) and the peak-to-peak amplitude was computed. The threshold value was defined as the lowest intensity able to evoke a response two times larger than the baseline amplitude.

Statistical comparisons were performed using the paired Wilcoxon test and Mann-Whitney, for independent groups. A probability of p < 0.05 was considered as significant. Results were expressed as mean ± standard error of the mean (SEM), unless stated differently.

### Micro-computed tomography imaging and three-dimensional reconstruction

Guinea pig heads with the implants fixed in place and preserved in 3.5%−3.7% formaldehyde buffered solution were scanned on a micro-computed tomography system (µCT, XtremeCT II, SCANCO Medical AG, Brüttisellen, Switzerland) using a defined protocol for a voxel size resolution of 17.0 μm (68 kVp, 1470 μA, 100 W).

**Figure 2:**
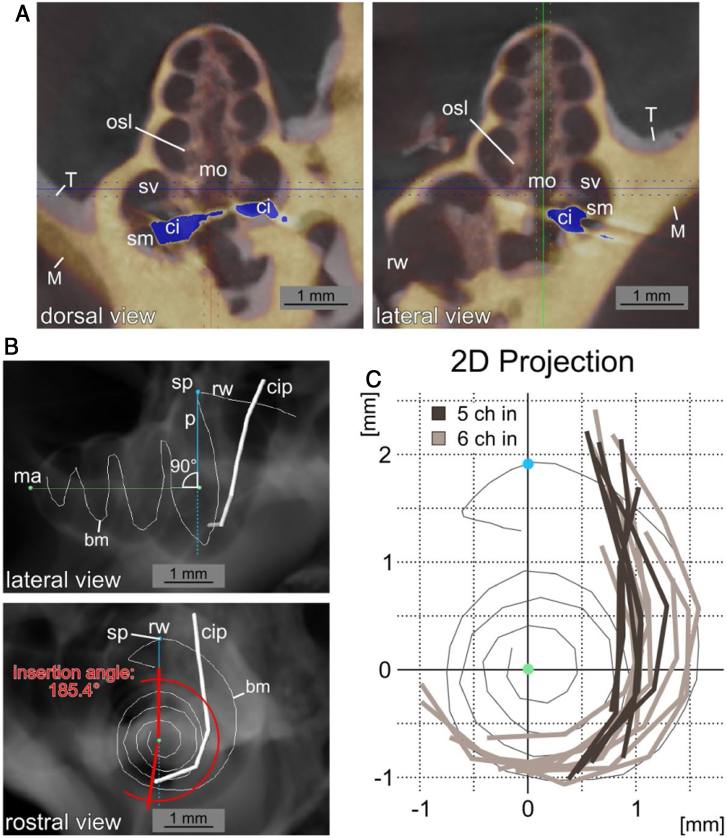
Insertion path reconstruction. A, Dorsal and lateral view of µCT data from an implanted cochlea (yellow, M) to the unimplanted template (grey, T) is depicted. The registration was performed to obtain the most agreement between the bony cochleae. The position of the CI with the µCT blooming artefacts is shown in blue (ci: cochlear implant; mo: modiolus; rw: round window; sm: scala media; sv: scala vestibuli). B, Lateral and rostral view of a digital radiograph view (from µCT data in Amira) of the non-implanted template with the reconstructed basilar membrane position (bm) and a superimposed exemplary cochlea implant position (cip) reconstruction. The images show the mid-modiolar axis (ma), the summit point of the basilar membrane reconstruction (sp) and the perpendicular to the modiolar axis (p). The measurement of the insertion angle (here 185.4°) is exemplified in the rostral view. C, Two dimensional projections in basal to apical direction of 15 cochlear implant positions from µCT data relative to the basilar membrane reconstruction. A pronounced difference in insertion depth between 5 contact (5Ch in, dark grey) and 6 contact (6Ch in, light grey) insertions is visible (round window and hook region on top, projection centered to mid-modiolar axis, compare B).

A high resolution µCT Dataset of an unimplanted, left cochlea was used to reconstruct the length of the basilar membrane along the midpoint between osseous spiral lamina and the attachment point of the spiral ligament in the 3D analysis software Amira (Version 6.0.1, FEI Corp., Hillsboro, OR, USA). The reconstruction included the hook region (curved basal region, adjacent to the round window). All implanted specimens were registered to this template in Amira. Right cochleae were mirrored to enable the registration. Then the coordinates of the contacts were measured. Thereafter, in order to turn this three-dimensional data (spiral shape), in a two-dimensional plane, values were rotated and projected in a rostro caudal two-dimensional representation of the cochlea. The projection was rotated to represent a view along the mid-modiolar rostro-caudal axis intersecting with the vertical perpendicular to summit of the basilar membrane before turning into the hook region (summit point). In this manner the length of the basilar membrane in percent, as described by Viberg et al.,^22^ the insertion depth (last contact as endpoint of insertion relative to basilar membrane length) and the insertion angle (relative to the perpendicular from summit point to the midmodiolar axis) were determined (Fig. 2).

The frequency-position of the electrode channels was also calculated, according to the species-specific Greenwood equation, in which 2.59 mm (∼14% of the cochlear length) distance inside the cochlea corresponds to 1 octave (2.59 mm/octave).

Electrophysiological data was analyzed from 24 ears in 14 guinea pigs and of those, µCT images were obtained from 20 ears in 10 animals. However, five datasets had to be excluded because of postmortem displacement of the CI or imaging problems; 15 ears of 9 animals were then included in the µCT analysis.

## Results

Extra-cochlear responses obtained with the silver ball electrode were evaluated as minimum thresholds at which a CAP was visible in the 2 − 32 kHz frequency range. Implantations could be anatomically reconstructed in 15 cochleae (Fig. 2) and showed different implantation depths in different animals (see below). Deepest implantations in optimal conditions may approach 270° insertion angle in the guinea pig. In the present experiments, no anatomical implantation trauma was detected in µCT images.

In the present experiments normal hearing animals were used; these are considered an extreme case that would not be implanted in clinical condition. However, this condition provides the optimal case for comparing the implantation trauma along the whole cochlear partition at high sensitivity.

**Figure 3:**
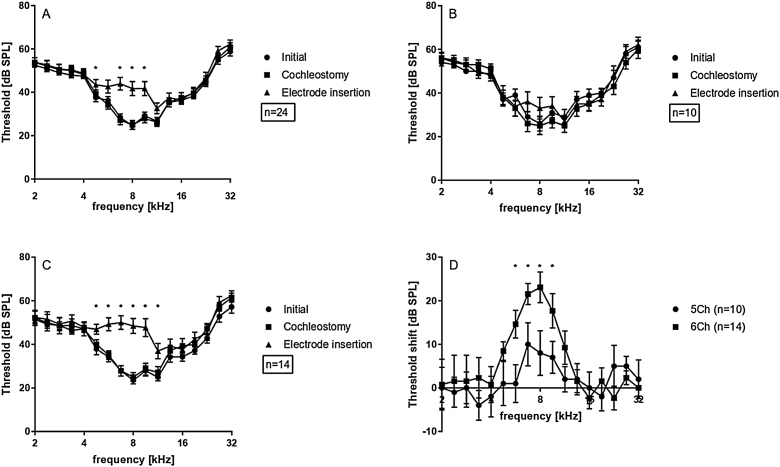
Extra-cochlear CAP thresholds: A, Grand average of all ears; B, 5 channel insertions; C, 6 channel insertions; D, Threshold shifts for 5 and 6 channel insertions; D, Statistical test: Wilcoxon signed rank; *p ≤ 0.05, error bars ± standard error.

Data from the 24 investigated cochleae are shown in Fig. 3A, displayed as mean and standard error of the mean (SEM). Immediately after cochleostomy, Threshold shifts (TS) were minimal or absent, and non-significant at all frequencies evaluated. However, in all cases electrode insertion caused some threshold shift. Significant TS occurred mainly at frequencies from 4.7–9.5 kHz. Responses to frequencies from 2.0 − 4.0 kHz were less affected by the implantation. Considering the length of the electrode (3.5 mm from apical to basal contact) and the Greenwood function,^22^ insertion induced shifts around the apex of the implant, in frequencies extending approximately 0.5–1 octave (or 1.3–2.6 mm) apically beyond the electrode coverage. This was confirmed by the implantation depths anatomically reconstructed in the 15 cochleae with µCTs images available (Fig. 2).

On average, TS reached at maximum 16.5 ± 2.7 dB, considering the 24 ears. This was a very mild hearing loss. The implantations as reported during surgery were subsequently divided to 5 contacts implantations (i.e. shallower implantations) and 6 contacts implantations (i.e. deeper implantations). In the 6 contact insertions (denoted in what follows as 6Ch) presented larger shifts (23.0 ± 3.5 dB, maximum TS) when compared to 5 contact insertions (denoted in what follows as 5Ch; 10.0 ± 4.5 dB maximum TS) (Fig. 3 B and C). TS were observed in the 4.7 and 5.6 kHz frequencies exclusively in the 6Ch group (not observed in the 5Ch group). Directly comparison of 6Ch to 5Ch groups revealed statistically significant differences in the frequency range of 5.7 − 9.5 kHz (Fig. 3D).

**Figure 4:**
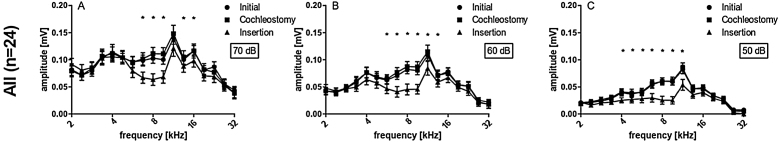
Extra-cochlear CAP amplitudes of all cases on average, at different stimulus intensities: (A) 70 dB; (B) 60 dB; (C) 50 dB. Statistical test, Wilcoxon signed rank; *p ≤ 0.05, error bars ± standard error.

Peak-to-peak amplitudes of suprathreshold CAPs were analyzed subsequently (Fig. 4), for different frequencies (2 − 32 kHz) and 50, 60 and 70 dB SPL. There were no differences between initial and cochleostomy conditions for neither stimulus level. Electrode insertion, however, induced a statistically significant decrease in CAP amplitudes at frequencies 6.7, 8.0 and 9.5 for 70, 60 and 50 dB level of stimulation when all cases were considered together (Fig. 4 A, B and C), mirroring the TS shown in Fig. 3.

**Figure 5:**
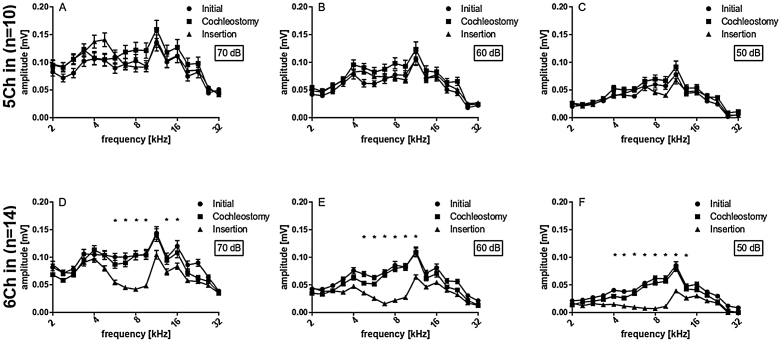
5 vs. 6 channel insertions. Upper graphs (A − C) depict the amplitudes obtained for 5 channel insertions with different stimulus intensities, while lower graphs (D − F) depict the amplitudes obtained for 6 channel insertions with different stimulus intensities, accordingly. 6 channel insertions present significant decreases in CAP amplitudes for different stimulus intensities, which are not observed in 5 channel insertions. Statistical test: Wilcoxon signed rank; *p ≤ 0.05, error bars ± standard error.

Subsequently we compared the deep and shallow implantations. With 5Ch implanted, we did not find statistically significant implantation effects on the amplitudes for all levels tested (Fig. 5 A, B and C). On the other hand, the 6Ch group showed amplitude changes covering a broad frequency range of 4 − 16 kHz (Fig. 5A − F).

**Figure 6:**
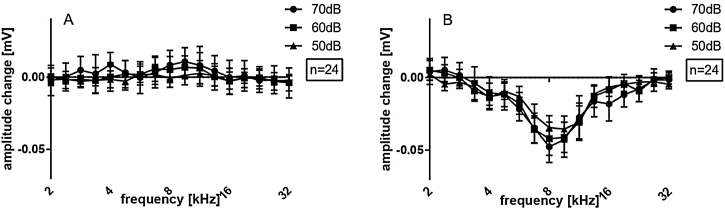
Extra-cochlear CAP presented as amplitude changes of all cases on average: (A) after cochleostomy; (B) after electrode insertion. Statistical test: Wilcoxon signed rank; *p ≤ 0.05 error bars ± standard error.

**Figure 7:**
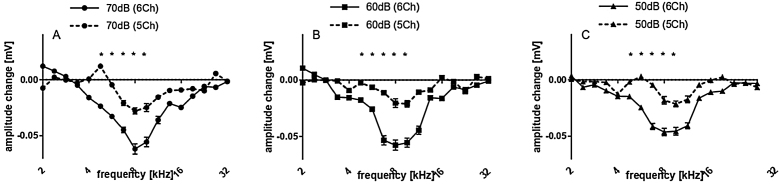
Amplitude changes of CAP after 5 and 6 channel insertions, at different stimulus intensities (70, 60 and 50 dB). Statistical test: Mann-Whitney; *p ≤ 0.05, error bars ± standard error.

Amplitude change for all cases together is evaluated in Fig. 6 for both groups pooled together. There were no significant amplitude changes detected after cochleostomy (Fig. 6A). After implantation, threshold shifts were found mainly from 5.6–11.3 kHz. When dividing the cases between 5Ch and 6Ch groups, larger amplitude changes and a broader frequency spectrum in the deeper insertion group (6Ch group) were identified (Fig. 7 A − C). Amplitude changes were particularly larger for the 6Ch group at 4.7 and 9.5 kHz.

From all insertions reconstructed in µCT the median lowest characteristic frequency reached by the implanted electrode was 8.04 kHz. The deepest insertion in our subset reached 5.51 kHz, whereas the shallowest insertion reached 9.96 kHz.

Implantations in 5Ch group covered less than 33% (average: 31,3%; ±1.1%) of the full basilar membrane length. On the other hand, the 6Ch group showed deeper implantations: more than 34% of the full basilar membrane length in every case (average: 37,1%; ±2.5%). The average insertion depth of the most apical electrode (Ch1) was 5340 ± 307 µm in 6Ch group vs. 4448 ± 290 µm in 5Ch group.

**Figure 8:**
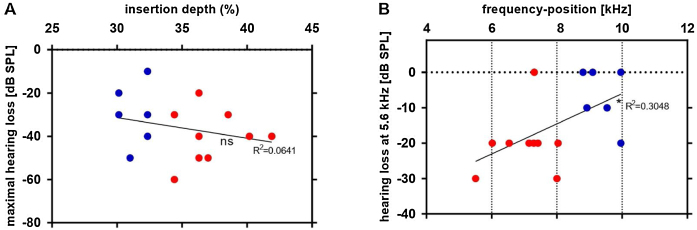
Linear regression between electrode depth estimated by µCT and the thresholds shifts after insertion: as compared to shallower insertions (blue dots), deeper insertions (red dots) correlate to greater maximal hearing loss (A). The frequency position calculated by Greenwood function of electrode contact 1 was correlated to hearing loss at 5.6 kHz (B). *p ≤ 0.05; ns: non-significant.

Finally, the electrode depth and its calculated frequency positions were related to the threshold shift caused by the implantation (post threshold – pre threshold). There was no significant correlation between the insertion depth and the maximal hearing loss Fig. 8A. A significant positive relation between the frequency position of electrode contact 1 and hearing loss at 5.6 kHz was observed (Fig. 8B). The 5.6 kHz is the lowest frequency in which a significant is detected and was therefore chosen. This result documents that there are other factors beyond implantation depth that are responsible for the largest hearing loss observed, but the previous data clearly show that the danger of hearing loss per se is higher if deeper implantations are attempted.

## Discussion

The present study evaluates implantation trauma in the context of CI surgery in normal-hearing guinea pigs. This exceptional condition, not accessible in humans, allowed comparing the implantation trauma specifically at different cochlear partitions. Furthermore, the experiments allowed relating the position of the electrode using anatomical reconstruction with a µCT.

This study demonstrated that it is possible to implant a hearing ear without hearing loss^23^ provided implantations avoided insertion till a resistance was felt (5Ch group; comparable to previous findings).^24^ Similarly, in human temporal bones it could be demonstrated that cochlear damage cannot be always detected by the surgeon because the related forces are below the sensitivity of the human hand.^25^ Avoiding the resistance should therefore be one goal of cochlear implantation.

Deeper electrode insertions had a larger impact on the inner ear function. The elicited tone-specific CAP indirectly reflects electrode position by its frequency specificity. TS and amplitude changes increased also in lower frequencies when the electrode reached cochlear regions in the 2nd half of the basal turn (beyond 180°). Despite this the results also showed that electrode insertion may be accomplished causing virtually no effect in apical-most frequency regions (< 4 kHz). Usually, electrophysiological interference spreads beyond electrode tip, towards frequencies represented in second cochlear turn. This may be the consequence of the damage that affects the basilar membrane or be due to a piston-type of effect when the scala tympani is filled out by the cochlear implant (as also seen previously).^24^

Electrode insertion could be accomplished in some cases with restricted hearing loss, keeping several frequency ranges unaltered. In this data series, we did not check for reversibility of the threshold shifts by withdrawing the electrode, as other authors^9^, ^10^, ^26^ did, which could help reveal the etiology of the amplitude shifts, as mechanical interference to basilar membrane.

It is interesting that the implantation trauma in the present cases involved mainly the electrode tip.^27^, ^28^, ^29^ Trauma in human temporal bones has been observed at the base of the cochlea due to implant buckling after resistance is encountered.^25^ We did not observe such damage here. Whether this is due to different anatomy of the guinea pig cochlea, due to dedicated implants for guinea pig with different mechanical properties or due to different angle in inserting the implant to the cochlea (the position of cochleostomy in guinea pig vs. human cochlea).

In some cases, we observed amplitude increases in some frequencies after cochleostomy or even electrode insertion, which could be due to perilymph leakage and contact to extra-cochlear electrode. This phenomenon could increase signaling because of direct intra-cochlear potential detection. Another possibility is that the effect of stiffening due to the electrode in basal turn may increase motion in more apical turns.^30^

ECochG response changes during CI are consistent with previous results^31^, ^32^, ^33^ showing smaller CAP amplitudes and higher thresholds, indicating cochlear damage. We did not address cochlear damage directly by means of hair cell counts or other histologic techniques, whereas we consider that our aim is not to elucidate the etiology of cochlear trauma. Direct comparisons between histology and electrophysiological data may be variable, as seen in other studies.^9^, ^19^ Previous studies have issued ECochG as a guide during cochlear implantation, identifying markers of mechanical damage and correlating with post-operative performance.^21^ Magnitude of CAP and CM amplitude loss can be correlated to the degree of histological damage caused by basilar membrane penetrations.^17^ Here we cannot provide the eventual explanation of the cochlear trauma. While several candidate mechanisms may be involved (direct mechanical damage to hair cells by lifting the basilar membrane; detachment of tectorial membrane from outer hair cells; interference with the potassium recycling or mechanical damage to the stria vascularis) and basilar membrane damage remains a possibility; the small effect size observed here likely indicates a predominant influence on the outer hair cells.

The scala tympani cross sectional area as a function of distance from round window was characterized by Ghiz et al.^34^ The electrode used in our study has a cross sectional area of 0.196 mm^2^ (0.5 mm diameter), with 4.5 mm maximum length. In guinea pigs, the cross-sectional area of the scala tympani abruptly diminishes towards apical in the first turn region (from circa 1.28 mm^2^ at the widest point of hook area to circa 0.29 mm^2^ at 5 mm round window distance).^34^ Our 5Ch cases have presumably reached ST in larger cross-sectional areas compared to our 6Ch insertions (700 micrometres longer), which reached narrower regions. The surrounding ST microenvironment in which the electrode is positioned is probably determinant of the differences encountered in our insertions. The narrower the region, the lesser amount of perilymph around and closer the electrode is to the organic signal source. Also, the possibility of basilar membrane contacts or even damage when the electrode advances further in ST is higher.

We are aware that damage to cochlear microanatomy and hearing loss after CI may occur weeks to months after surgery,^35^ which is a main concern when hearing preservation is a goal. Many factors are involved, including inflammation, fibrosis, hydrops, mechanical effects (movement of the electrode) and even idiopathic factors.^36^ These events may take place with or without clearly observable histologic and/or radiologic damage. We did not observe in our µCT sequences gross trauma over the spiral osseous lamina or other transgressions in the electrode path inside the cochleae. Nevertheless, µCT has limited accuracy and resolution for detecting trauma in membranous structures, such as the organ of Corti and stria vascularis.

Our data suggests that shorter electrodes have lesser impact on cochlear physiology, on an intraoperative setting. A present dilemma is that although short electrodes seem safer, they may offer less performance to CI users, mainly but not only in the context of electroacoustic stimulation. Ideal placement and positioning are therefore desirable. Intraoperative events in CI surgery are potentially crucial to surgical decisions, and electrophysiology may be a tool to indicate the choice of an electrode over another. Finally, the human cochlear anatomy is highly variable.^37^, ^38^ The present study suggests that predictions of the implantation process may prevent too- deep implantations, and thus precise planning in surgery, using imaging,^39^, ^40^ supported by cochlear models^41^, ^42^, ^43^ and electrophysiology^24^, ^44^, ^45^ might help to prevent cochlear trauma.

## Conclusion

In this guinea pig model of cochlear implantation, cochleostomy induced little or none impact in the extracochlear compound action potentials. Deeper insertions led to greater hearing loss, including the cochlear region apical to the implant position. The hearing loss was restricted to a predicable frequency range around the tip of the cochlear implant.

## Funding

J.S.C.A received a scholarship from CAPES Foundation, Ministry of Education of Brazil, Brasília – DF 70.040-020, Brazil.

## Authors' contributions

José Santos Cruz de Andrade and Peter Baumhoff contributed equally to this work: data acquisitions and analysis, writing and reviewing the paper. Andrej Kral made data analysis, writing and reviewing the paper. Oswaldo Laércio Mendonça Cruz and Thomas Lenarz both made writing and reviewing the paper.

## Conflicts of interest

The authors declare no conflicts of interest.
